# Overseas Care and Cancer Survivorship in Small Island Developing States of the Eastern Caribbean: Protocol for a Multicountry Mixed Methods Study (CaSIDEC Study)

**DOI:** 10.2196/90930

**Published:** 2026-07-31

**Authors:** Aviane Auguste, CaSIDEC Study Group, Eunetta Bird

**Affiliations:** 1 Department of Epidemiology Biostatistics and Occupational Health, School of Population and Global Health, McGill University, 2001 McGill College Avenue, Montreal, QC, H3A 1G1, Canada,; 2 Vaughan A. Lewis Institute for Research and Innovation (VALIRI), Sir Arthur Lewis Community College, Castries, Saint Lucia; 3 See Acknowledgements

**Keywords:** cancer, survivorship, medical tourism, treatment, lifestyle, social support, health systems, small island developing states, Caribbean, Saint Lucia

## Abstract

**Background:**

Small island developing states (SIDS) are home to approximately 65 million people worldwide. SIDS have some of the highest rates of cancer mortality burden in the developing world. Disparities in cancer mortality could be attributed to reliance on services overseas, which is a common feature across SIDS due to limited resources for comprehensive cancer care on the islands. However, overseas travel for cancer care remains largely understudied in SIDS, and epidemiological data on the implications on cancer survivorship are lacking.

**Objective:**

We aim primarily to map patterns of overseas travel for cancer care among cancer survivors in Caribbean SIDS and examine the lived experiences of cancer survivors and caregivers. Additionally, we will explore how overseas travel may affect key domains of cancer survivorship.

**Methods:**

We will conduct a multicountry, cross-sectional study using a convergent mixed methods design. We will establish a sample of 900 cancer survivors residing in the islands of Antigua, Dominica, Grenada, Saint Kitts, Saint Lucia, and Saint Vincent. Eligible participants will be adult cancer survivors who have accessed health services in their island of residence due to cancer. Sites for recruitment will be cancer support groups and hospitals. Surveys will be conducted face-to-face. For every country visited for diagnosis and/or treatment, we will record the services accessed and motives for the choice of country. We will compute the proportions of patients traveling for cancer care by country of destination. We will use regression models to explore associations with time to treatment and lifestyle factors (after treatment). In-depth interviews (n=24) and focus groups (n=54 participants) will be conducted with cancer survivors and caregivers to learn about overseas travel with an emphasis on social support systems. Open-ended questions and prompts will assess aspects of the day-to-day logistics and travel arrangements and experience living overseas. We will use an inductive thematic analysis approach to identify and interpret patterns.

**Results:**

Planning and development to implement the logistics for data collection were started in February 2023. We started with pilot testing in September 2024, and we have recruited 113 cancer survivors as of May 31, 2026. We envisage active patient recruitment and data collection until March 31, 2029, across all participating countries.

**Conclusions:**

This is the first multicountry study on overseas travel on cancer care in Caribbean SIDS. Our work will produce evidence that will inform recommendations to fill performance gaps in transnational systems for cancer care in the Eastern Caribbean that will improve patients’ lives. It is an important first step to developing data resources for high-quality research in understudied SIDS.

## Introduction

### Cancer Burden in Small Island Developing States

Populations that are geographically isolated and resource constrained (GIRC) face heavier cancer burdens than those in more populous and affluent settings [1,2] due to critical performance gaps in cancer control in these GIRC settings. In GIRC settings, the proportion of patients presenting with advanced-stage cancer is higher and is driven in part by low health literacy and insufficient opportunities for cancer screening. Data on staging at diagnosis are scarce, but some data on breast cancer are available. A study reported the distribution of breast cancer cases by stage in the Caribbean. Only 10.6% to 11.2% of Caribbean women diagnosed with breast cancer present at stage 1, compared with 48% of women in the United States [3,4]. The few numbers of oncologists and pathologists available for patient care in these settings also hinder access to life-saving cancer treatment. Little scholarly work exists on cancer control in GIRC settings to address these gaps [1,5,6].

Among GIRC populations, small island developing states (SIDS) are a subset that make up 65 million people worldwide [7]. SIDS have some of the highest rates of cancer mortality relative to other world regions comprising mostly low- and-middle-income countries where patients with cancer experience similar structural barriers to early detection and care. Compared to Africa and Southeast Asia (87.4 and 93.1 per 100,000 inhabitants, respectively), age-standardized mortality rates for cancer are higher in small islands from the Caribbean and Polynesia (vs 99.2 and 118.9 per 100,000 inhabitants, respectively) [8]. Disparities in cancer mortality could be attributed to heavy reliance on care services overseas, which is a common feature across SIDS [9,10] due to limited resources for comprehensive cancer care on the islands [11].

### Overseas Travel for Cancer Care and Survivorship

Referral for cancer care overseas in SIDS is often perceived as an opportunity to access better quality services compared to those available locally [5,12]. For people diagnosed with cancer in SIDS (cancer survivors), overseas care may provide access to more specialized services, more advanced medical technologies, better oncology expertise, and a higher standard of care. However, an overseas referral is a complex process in SIDS and is characterized mostly as paid out of pocket and as travel both within the region and toward larger and more developed countries [1]. This reliance on overseas travel in SIDS also introduces important considerations where cancer survivors face a trade-off between logistical challenges and access to better quality services. The potential benefits from accessing these services overseas may be compromised by time delays. Time delays may occur for a myriad of reasons (system, provider, or patient related), such as having to mobilize resources to finance the stay overseas, long decision-making intervals, waiting for visa approval, and other logistics [12]. The consequences for patient outcomes may include stage shifts to more advanced disease that are more difficult to treat and have a worse prognosis. Hence, these time delays from overseas travel could considerably reduce patients' quality of life and probability of survival. Little is known about how the process of overseas referral from SIDS affects subsequent delays in the care continuum.

In addition to treatment delays, survivorship care in this context is an issue. The incidence of cancers is expected to increase in SIDS in the coming decades [13]; however, small island health systems are currently not prepared to satisfy the growing demand in survivorship care [5]. Survivorship care is aimed at preventing cancer recurrence and secondary cancers and improving quality of life through healthy lifestyles [14]. Healthy lifestyles can reduce the risk of mortality or disability from cancer and treatment-associated problems, such as cardiometabolic and lung disease, bone loss, eye and hearing changes, and lymphedema [15-20]. Regular physical activity (PA) and healthier diets can contribute to better long-term health outcomes among patients with cancer [21]. Management of these late treatment effects, follow-up care, and health promotion services is underdeveloped in Eastern Caribbean SIDS [1]. Consequently, long-term outcomes for survivors who traveled for care may be better than those who did not due to additional health promotion and resources offered by providers overseas compared to those on the islands [1,22,23]. Disparities in mortality and long-term disabilities among working survivors deprive the Caribbean of productive workers who contribute to much-needed economic growth [24]. However, Caribbean studies on lifestyle factors among cancer survivors are scarce. Previous studies on lifestyle have focused largely on the period before diagnosis [25], which does not reflect lifestyle later during the disease trajectory [26]. Furthermore, the potential disparities between survivorship care done locally and overseas have not been measured.

Social isolation is another issue that may arise from overseas travel for cancer care. This could be counterproductive, leading to worse health outcomes [27,28]. Social support is defined as a network of family, friends, neighbors, and community members that is available in times of need to give psychological, physical, and financial help [29]. The link between social support and improved quality of life is well established [30-32]. A previous study on patients with breast cancer showed that having social support mediates the choice of coping strategies toward positive reframing, which leads to better emotional well-being [31]. The role of social support in overseas medical travel in the Caribbean is unclear, but the putative benefits on patient outcomes from better health services overseas may be mediated by social support. There is a need to understand and explore how social support influences cancer care in the Caribbean. To date, no investigation has been conducted comparing social support systems of Caribbean cancer survivors treated overseas with those treated on the islands. Preliminary data showed that Saint Lucian survivors depended highly on support from family for a positive cancer experience and may be particularly vulnerable during overseas travel when they are forced to separate from their family [33].

Ultimately, the literature supports the idea of potential negative consequences of overseas cancer care on several survivorship domains, such as timely treatment, social support, and lifestyle [34]. However, the overall influence on survivorship in the short term and long term remains unclear in SIDS, where medical travel is common. Hence, this knowledge gap motivates the need to learn more about overseas cancer care in SIDS.

### Cancer Care Gaps in the SIDS of the Organization of Eastern Caribbean States

The SIDS that make up the Organization of Eastern Caribbean States (OECS) are particularly susceptible to system-related cancer mortality [35] because of limited access to specialized resources for oncology and management of patients with cancer in these islands. Limitations in resources include radiotherapy facilities, advanced diagnostics, and oncology-trained personnel, thus creating great reliance on health services outside the country of residence. This limited access to services locally is further compounded by underdeveloped health policies for cancer care that could contribute to more equitable access and patient safety when overseas referrals are required [36]. Current referral practices for overseas care in the OECS may expose patients to unnecessarily long waiting times [37]. Indeed, in the absence of adequate policy, travel restrictions resulting from external shocks (such as natural disasters and pandemics) and suboptimal processes across the care continuum may continue to contribute to higher cancer mortality.

Data on the cancer burden in the OECS region are limited [2,38]. Cancer represents a leading cause of mortality in SIDS across multiple anatomical sites. Only Saint Lucia has estimates available from the GLOBOCAN database. On the basis of these 2022 age-standardized incidence rates, we know that the most common cancers for Saint Lucia are prostate (93.9), breast (51.7), cervical (15.7), and colon (12.3) [2].

The OECS nationals residing in the islands of Antigua, Dominica, Grenada, Saint Kitts, Saint Lucia, and Saint Vincent (~600,000 inhabitants) belong to the same economic union, have freedom of movement, and a common political agenda regarding cancer control [39]. Chemotherapy and other pharmaceuticals for these 6 islands are procured by the OECS Commission. On the one hand, all these islands have similar capacity in terms of cancer diagnosis, general surgery, and medical oncology [1]. On the other hand, there are a few differences in terms of health services offered; for example, Antigua had radiotherapy (2016‐2023), and the oncology units in Saint Kitts and Saint Vincent were more recently set up compared to the other OECS member states. In addition, preferences for travel destinations for oncology care are often linked to geographical and sociocultural proximity with those countries.

Despite the socioeconomic cohesion in the OECS subregion, there is limited accessible information on the availability of diagnostic and treatment services across the region. This makes it difficult for patients and providers to make informed decisions about care options. Similarly, having this information could be motivating for providers and policymakers from the OECS to decide on what resources could be leveraged among member states and internationally to improve access for their patients. Therefore, a multicenter approach for epidemiological studies within the OECS will not only overcome sample size limitations inherent to SIDS but also help ensure greater generalizability and capacity for the implementation of new regional cancer control policies within the OECS.

### Gaps in Policy-Relevant Evidence

To improve mortality rates and cancer survivorship, policymakers need to determine priority areas for better cancer care. This process includes deciding between improving access to cancer services from overseas and developing infrastructure and services on the islands. These crucial decisions should be based on scientific evidence examining itineraries for medical travel, characteristics of travelers, and the services being accessed overseas. However, overseas travel, with regard to cancer care, remains largely understudied in SIDS [1,3,40], and vital epidemiological data on the impact on cancer survivorship are lacking.

Recent review articles have presented the treatment landscape of Caribbean SIDS [1,3,36]. One review emphasized that models of cancer care from high-income countries are not always appropriate for the Caribbean context [1]. Another review reported that approximately hundreds of patients from the islands of Grenada, Dominica, and Saint Lucia receive care every year in Martinique, a developed French-Caribbean island [41]. These reviews were pivotal first works for the Caribbean, but like most studies on this topic, they were lacking patient-level data (eg, characteristics and lived experience) [1,3]. Our preliminary work from the DCAP study (Description of the CAncer health services: diagnosis and treatment Pathways) showed that in Saint Lucia, a little more than half of patients with cancer travel for cancer diagnostic services and treatment [5,33]. However, the DCAP study lacked the sample size to make strong conclusions to support these complex policy questions. Other studies from the Caribbean do not address the lived experiences, treatment times, lifestyles, and characteristics of patients seeking care overseas.

### Research Aims and Hypotheses

#### Overview

CaSIDEC is an acronym for “Cancer in SIDS of the Eastern Caribbean.” Our overarching aim is to explore how overseas travel for cancer care may affect key domains of cancer survivorship [34]. Survivorship will be our long-term focus and goes beyond survival as commonly measured in clinical trials (ie, time to death from diagnosis) and will include measures on how individuals experience life with cancer. In this study, overseas care will be considered as any travel across international country borders (including the Caribbean), regardless of the payment mechanism (eg, out of pocket or government subsidized).

As a first step, we wish to contribute toward building research capacity in SIDS. The CaSIDEC study aims to generate foundational evidence on transnational cancer care pathways and survivorship in SIDS of the Eastern Caribbean. Given the limited availability of standardized epidemiological data in this region, we wish to establish data resources, measurement approaches, and analytic frameworks necessary to support future hypothesis-driven and causal research on this topic.

**Figure 1.**
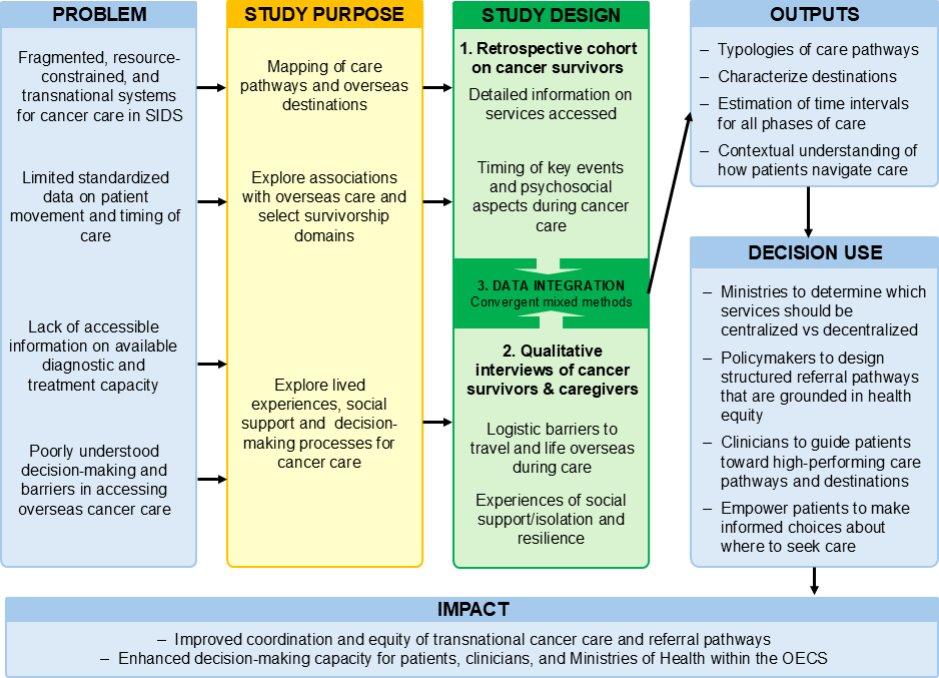
Conceptual logic model of the CaSIDEC study. The diagram starts from problems that map onto the study purpose and a study with a convergent mixed methods design. These approaches will generate outputs insights into patient experiences. The outputs are intended to inform decision-making by Ministries of Health, clinicians, and patients, ultimately improving coordination, equity, and evidence-based planning of transnational cancer care. Color coding distinguishes study components (in yellow or green) from broader contextual elements and applications (in blue). CaSIDEC: Cancer in small island developing states of the Eastern Caribbean; OECS: Organization of Eastern Caribbean States; SIDS: small island developing states.

In terms of impact, the data from this study will guide decisions at the patient, provider, and policy levels. It will notably inform decisions on how overseas referral pathways should be organized across the OECS subregion. Figure 1 presents a conceptual logic model illustrating the study framework, including the link between problems, knowledge gaps, and intended impact of the CaSIDEC study.

#### Primary Aims

Our primary aims are to (P1) map patterns of overseas travel for cancer care among cancer survivors of 6 member states of the OECS and (P2) explore the lived experience of cancer survivors and caregivers and how overseas travel for care affects their social support systems.

We anticipate that a substantial proportion of cancer survivors travel outside of their resident country for cancer treatment. We expect certain destinations to be more frequented among cancer survivors by individual characteristics (cancer site, stage, and country of residence). We also expect that overseas care may be associated with both challenges and adaptations in social support systems, as explored qualitatively through survivor and caregiver accounts.

#### Secondary Aims

In addition to our primary research aims, we will leverage these data to explore the role of overseas care in 2 survivorship-related domains. Specifically, we will attempt to investigate potential associations between overseas travel for cancer care and (S1) treatment delays and (S2) lifestyle factors in the months following active treatment.

We expect there to be differences in time to treatment and lifestyle behaviors between cancer survivors who were treated on island and those treated overseas. Given the exploratory nature of the secondary objectives, data from these analyses will be interpreted with caution.

## Methods

### Study Design and Sampling

CaSIDEC is a multicountry observational study. Our study will use a convergent mixed methods design. Quantitative and qualitative data will be collected and analyzed independently and then interpreted using joint displays [42]. The quantitative component will focus on mapping patterns of overseas care, while the qualitative component will explore lived experiences of cancer survivors and caregivers, particularly in relation to social support. This protocol was prepared in accordance with the Observational Quantitative and Qualitative Study checklists and SPIRIT (Standard Protocol Items: Recommendations for Interventional Trials) reporting checklist (Checklist 1).

The CaSIDEC study builds on prior work conducted in Saint Lucia published as a pilot under the name “DCAP study” [5]. The DCAP study demonstrated the feasibility of collecting detailed retrospective data from a small sample of cancer survivors (n=50; Table 1). In addition, this pilot study informed this study design and instrument development and oriented the focus on overseas travel for cancer care [5]. The details on the study design and data from the DCAP study are described in detail elsewhere [5].

**Table 1.** Characteristics of patients included in the DCAP^a^ pilot study (N=50), Saint Lucia (West Indies), 2019 to 2020.

Characteristics	Values, n (%)
Cancer site	
Breast	26 (52.0)
Female pelvis^b^	10 (20.0)
Prostate	9 (18.0)
Other^c^	5 (10.0)
Sex	
Male	13 (26.0)
Female	37 (74.0)
Age at diagnosis (y)	
<50	15 (30.0)
50‐65	26 (52.0)
>65	9 (18.0)
Survivorship^d^ (y)	
<7^e^	35 (71.4)
7‐10	9 (18.4)
>10	5 (10.2)
Education level^d^	
Primary	16 (32.7)
Secondary	15 (30.6)
Tertiary	18 (36.7)

a DCAP: Description of the CAncer health services: diagnosis and treatment Pathways.

b Cervix, endometrium, ovary.

c Colon, parotid gland, and leukemia.

d Missing data for 1 patient.

e Four patients surveyed <3 mo of cancer diagnosis.

**Figure 2.**
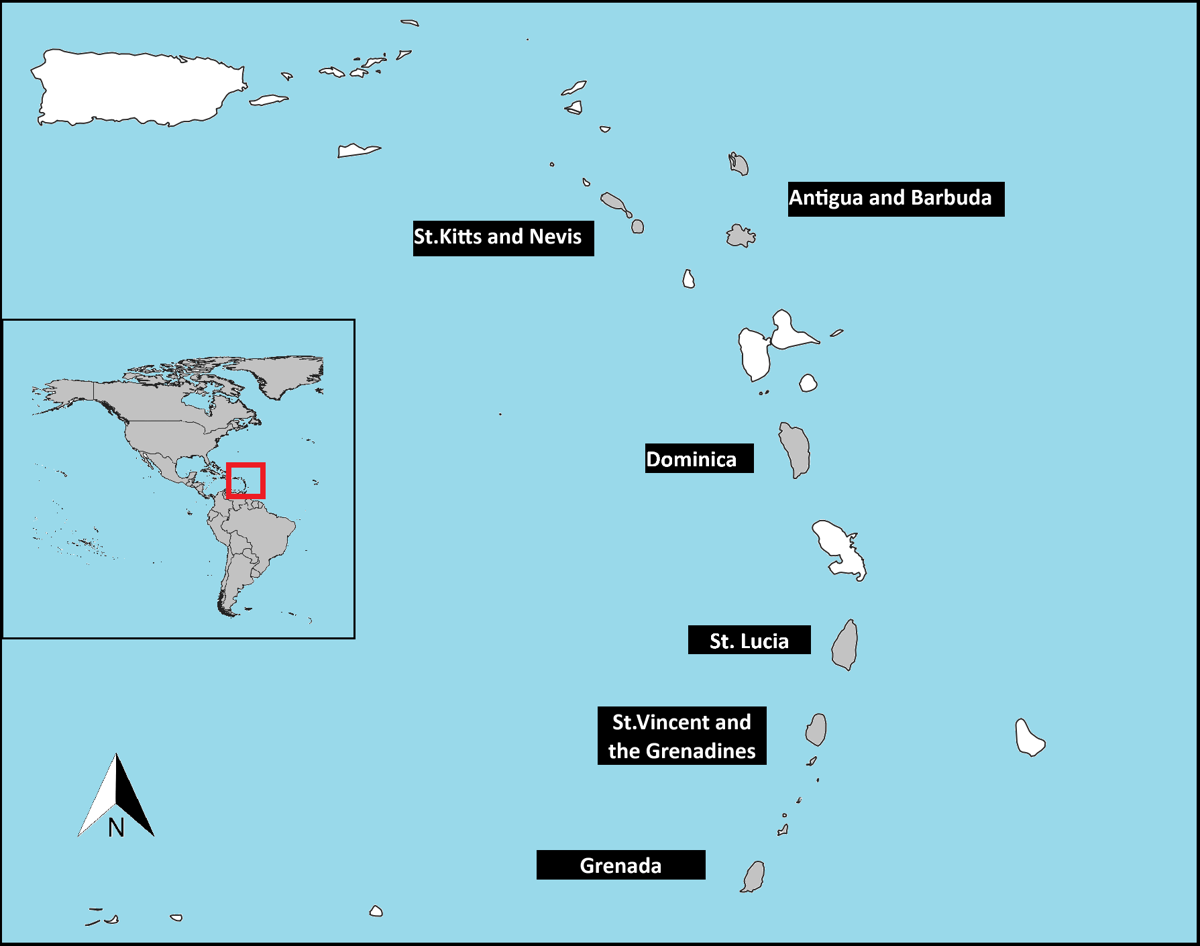
The 6 island states in the Eastern Caribbean covered by the CaSIDEC study: Antigua and Barbuda, Dominica, Grenada, Saint Kitts and Nevis, Saint Lucia, and Saint Vincent and the Grenadines. The inset map shows the location of the Eastern Caribbean within the Americas. Maps were created using QGIS and Natural Earth data (public domain). CaSIDEC: Cancer in small island developing states of the Eastern Caribbean.

For this study, we will establish a cross-sectional sample of 900 cancer survivors, including data to retrospectively reconstruct care pathways and ascertain present-day variables. Eligible participants will be cancer survivors residing on the islands of Antigua, Dominica, Grenada, Saint Kitts, Saint Lucia, and Saint Vincent (Figure 2). Additionally, participants will be aged ≥18 years; able to communicate in English, Creole, or local dialect (without cognitive impairment); have a diagnosis of cancer (any cancer site, histology, and diagnosis year), and have accessed health services in their island of residence due to cancer. Participation in the CaSIDEC study will include written authorization to access patients' data from medical records in health care institutions.

For aim S2 (lifestyle after cancer), eligible participants will be a subset of patients from aim S1 who have already finished their initial active treatment at least 3 months before the time of the survey. We will investigate lifestyle factors within 3 months of ending active treatment. Participants may be surveyed at any point after their diagnosis. Thus, those who finished treatment after 3 months will have two measures for lifestyle: (1) retrospectively at 3 months after treatment and (2) at the time of the survey.

We will aim to recruit a sample of cancer survivors that approximates the distribution of cancer survivors in the OECS by age, sex, district of residence, income, and cancer site. As there are no population-based cancer registries in these SIDS, the target distribution for sampling of cancer cases for each island will be developed from data from the World Health Organization reports [43], GLOBOCAN, hospital registries (where available) [38], cancer support groups, and medical assistance programs. Every 6 months during data collection, we will generate the proportions of these characteristics in the study sample by island. This routine verification will identify the patient profiles that are over- or underrepresented compared to the projected target sample. We will then adjust the recruitment strategy to ensure that our sample conforms to the target distribution.

### Recruitment

#### Overview

In the absence of established, population-based cancer registries in the OECS member states under study, we will use a nonprobability sampling framework. This framework comprises 2 distinct pathways for patient accrual that are meant to prioritize wide reach and diversity. Snowball sampling will be used throughout the study to identify prospective participants [44]. Sites for recruitment will be cancer support groups, local hospitals, and oncology clinics in both the public and private sectors.

#### Purposive Sampling

We will purposively recruit eligible volunteers from the general public, the community, and civil society to capture a broad group of long-term, active cancer survivors. Recruitment will be advertised through social media, public broadcasting media, and the large mailing lists from our cancer support groups. We will also leverage the local medical fraternity organizations and the Ministries of Health to assist in advertising participant recruitment. The cancer support groups will identify eligible participants among their group membership. Where possible, we will recruit cancer survivors (including nonmembers) during cancer advocacy activities organized by the cancer support groups (eg, health fairs). Patients at health care establishments will be recruited during opportunistic cancer navigation assistance by a representative from a support group.

#### Consecutive Sampling

**Figure 3.**
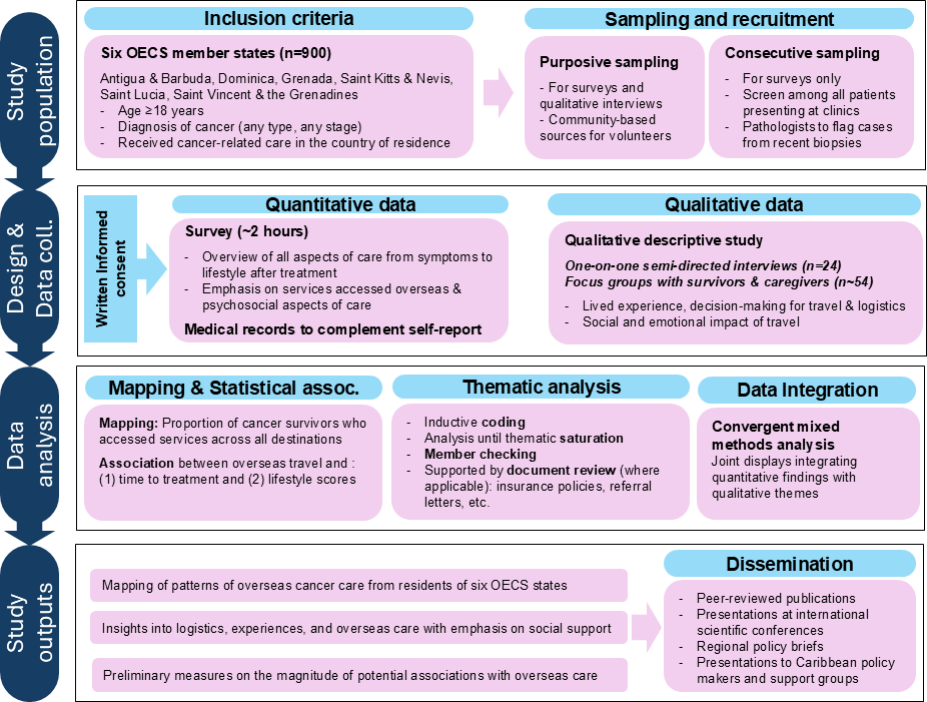
Study flow diagram for the CaSIDEC study. CaSIDEC: Cancer in small island developing states of the Eastern Caribbean; OECS: Organization of Eastern Caribbean States.

We will use consecutive sampling to recruit all eligible patients who present at clinical settings during the study. Local clinicians will be trained to actively inform and recruit eligible patients from their practice during routine care. We will work with oncologists and other clinicians to identify and recruit recently diagnosed and follow-up patients through contact registers maintained by the clinical team. Additionally, pathologists will flag eligible cases based on recent tumor biopsies. Figure 3 presents the overall study process, including recruitment, data collection, analysis plan, and dissemination plan.

### Data Collection

Once consented, eligible participants will be contacted by a trained interviewer for a face-to-face survey. Face-to-face is preferred, as this reduces issues related to the interpretation of different terms and time intervals that are commonly associated with self-completion questionnaires in similar research [45]. Our study will be guided by the “Model of Pathways to Treatment” from Walter et al [45,46] to inform the study design and data analysis. This model provides a structured framework to characterize distinct phases, key events, and time intervals along the cancer care continuum. This model will notably inform the reconstruction of diagnostic and treatment pathways. Variable definitions will follow those from the model and will strengthen comparability with other studies.

presents a summary of measures for the quantitative survey that forms part of the CaSIDEC study. We will ascertain sociodemographics, such as education level, private medical insurance, income, hot water at home, employment or education, and clinical characteristics, such as cancer stage at diagnosis and comorbidities (Table 2).

**Table 2.** Summary of measures for quantitative survey part of the CaSIDEC^a^ study. Variables collected for primary aim 1 (aim P1) and secondary aims 1 and 2 (aim S1 and aim S2) are shown.

Domain	Variables and constructs
Sociodemographics^b^	Age, sex, ethnicity, address Private health insurance coverage Education, income, current employment Household items and equipment
Health status and tumor^c^	Cancer site, stage, diagnosis year Comorbidities diagnosed before cancer
Self-appraisal and help-seeking^b^	Experience with seeking medical help before cancer Symptoms Reaction to symptoms
Investigation of symptoms^c^	Presentation to health care providers Contents of consultation (advice and physical assessment) Tests and treatments prescribed Referrals for investigation and treatment Opinion on quality improvement
Diagnosis announcement	Health care provider making diagnostic announcements Patient’s perception of diagnostic information provided Opinion on quality improvement
Diagnosis tests^c^	Tests performed and turnaround time Result of tests
Cancer treatment^b^^,^^c^	Treatment initiation and modality Palliation and symptom control Pain and late treatment effects Comorbidities after cancer diagnosis
Care overseas^c^	Travel destinations for diagnosis/treatment Services accessed overseas Motivations for overseas care Connections to other countries (family and friends)
Psychosocial and supportive care	Consideration of personal circumstances Relationship with health care providers Participation in cancer support groups Supportive care services Funding of cancer care
Post-treatment follow-up	Routine follow-up Health promotion
Overall perception of experience	Quantitative rating Free response on overall experience living with cancer
Lifestyle post-treatment^b^^,^^d^	Dietary habits Hot drinks (tea and coffee) Substance use: tobacco, alcohol, and cannabis Physical activity^e^ Anthropometrics: height, weight, waist circumference

a CaSIDEC: Cancer in small island developing states of the Eastern Caribbean.

b New subquestionnaire or significant changes made to an existing subquestionnaire relative to the DCAP study (Description of the CAncer health services: diagnosis and treatment Pathways).

c Plan to complement with data from medical records.

d Diet and physical activity based on the 3-month period preceding the survey.

e Based primarily on the Short Questionnaire to Assess Health-Enhancing Physical Activity (SQUASH).

We will also collect detailed information from all consultations with health care providers (HCPs), from first presentation to initiation of active treatment, including clinical investigations for cancer, specialty of the HCP, location of consultation, scheduling and date of consultation, symptoms, tests and treatments prescribed, referrals, scheduling of review appointments, and suggestions for improving the described management. For each diagnostic test, date, laboratory name and location, turnaround time, and the finding (whether a test revealed suspicion of cancer) will be ascertained. All treatment modalities will be recorded, including natural or alternative remedies. For each modality, information from the first and last time it was administered (type, date, specialty of physician, location, and country) will be recorded. We will collect information on psychosocial support from loved ones and providers and supportive care services accessed.

Funding modalities, fundraising experience, and the associated psychological burden will be ascertained. We will ascertain access to palliative care, management of pain, and late treatment effects (eg, lymphedema). Modalities for posttreatment follow-up such as provider specialty, frequency of visits, tests, and health promotion will be collected during the interview. Participants’ personal appraisal of their experiences for major events will be ascertained throughout the interview using a *Likert scale* and open-ended questions [47]. For every country visited for diagnosis and/or treatment, we will record the services accessed and motives for the choice of country (personal or HCP preference vs lack of infrastructure).

We will use a Food Frequency Questionnaire based on the National Cancer Institute-Food Frequency Questionnaire [48]. Other traditional or alternative medicines consumed will be ascertained. Special attention will be given to tisanes from local plants, such as soursop (*Annona muricata L*.) and papaya (*Carica papaya L*.), which are consumed by cancer survivors from the Caribbean [5] and have anticancer properties [49,50]. Moderate- to vigorous-intensity PA will be self-reported by the validated SQUASH questionnaire (Short Questionnaire to Assess Health-Enhancing Physical Activity) [51]. BMI at the time of the interview will be ascertained using a scale and stadiometer. Waist circumference will be measured with a measuring tape by a trained field investigator. Self-report will be used for retrospective measures. We will also assess tobacco status, alcohol drinking, and cannabis use.

We will collect clinical data from the patients’ medical records from the various health care establishments (private and public) and medical laboratories on the islands. Data from medical records will help prevent issues related to data completeness and misclassification bias from self-report. We will abstract specifically clinicopathological characteristics of the cancer (anatomical site; tumor, node, and metastasis (TNM) staging, and histology), date of laboratory results, and cancer treatments.

### Statistical Analysis

Findings for the quantitative analyses will be interpreted as descriptive patterns and exploratory associations to develop hypotheses to study causal effects in future studies. To map and characterize patterns of overseas care (aim P1), we will compute the proportion of cancer survivors who traveled overseas for diagnosis and treatment among the entire sample. The proportion of overseas travel will be computed as well by cancer site, OECS country, period of diagnosis, and type of diagnostics and treatment accessed. Where possible we will characterize overseas travel by region (Caribbean, North America, South America, Europe, and other) and by destination country.

We will use multivariable Cox regressions as our primary model to estimate time from diagnosis to first treatment, as a function of overseas travel for treatment. Overseas travel will be treated as a binary variable (travel for treatment vs ref=treatment exclusively on the islands). Medical records data will be used to calculate the time from diagnosis (biopsy date) to treatment (first treatment date). To better interpret the potential clinical impact of our results, we will also operationalize the time to treatment variable as the number of days from recommended benchmarks based on scientific evidence (eg, UK’s National Health Service).

We will calculate overall lifestyle scores based on cancer prevention recommendations from the World Cancer Research Fund and American Institute for Cancer Research [52]. Quantitative cutoff points will be used to determine adherence to the different components from the guidelines. The quantitative cutoff points correspond mostly to values that significantly influence risk of cancer, noncommunicable diseases, and all-cause mortality [52,53]. We will use linear regression models to estimate the association between country of treatment (overseas vs on island) and healthy lifestyle. For these models, a 1-SD increase in each lifestyle score will be calculated to allow comparability between the scores. To achieve more clinically relevant interpretations, the scores will be dichotomized based on cutoff points determined by recommendations based on reducing all-cause mortality among cancer survivors [52]. The association with this variable will be estimated from proportionality ratios and their 95% CIs using multivariable log-binomial regressions.

All models will include age at diagnosis, cancer site, stage at diagnosis, gender, country of residence, socioeconomic status, and years since diagnosis (minimally adjusted). Models for aim S2 will be further adjusted for treatment type, comorbidities, and health promotion accessed. We will also consider using multiple imputations to manage missing data. We will also use directed acyclic graphs to help identify potential confounders [54]. An a priori alpha level of .05 will be used to determine statistical significance. We intend to analyze all participants together to maximize statistical power. However, we acknowledge that treatment waiting times and lifestyles may vary according to certain innate characteristics such as treatment protocols specific to cancer sites and country of residence, cultural differences, treatment location, and patients’ gender.

Sensitivity analyses will be performed to assess the impact of these potential variations on results and the study’s robustness. We will examine effect modifications and perform subgroup analyses by covariates listed in the minimally adjusted model. To minimize recall bias, we will systematically conduct analyses restricted to patients who were diagnosed more recently (aim S1: ≤12 mo, 5 y and aim S2: ≤24 mo). We will introduce the country of residence as a covariable or as a random effect in the regression model. Where possible, we will also perform analyses by country to highlight country-specific features.

### Power Calculation

Estimates for 2022 in Saint Lucia show a 5-year prevalence of 1119 cases and 449 new cases per year [2]. We anticipate a response rate of 75% as per the pilot study. Hence, we should be able to enroll 839 prevalent cancer cases in Saint Lucia alone. Despite the 5 other countries having smaller populations (54‐110,000), we assume that the other countries will have fewer cancer cases. During our recruitment period, we anticipate ~3000 eligible cases after adjusting prevalence estimates for country population size. All 6 countries altogether will ensure a substantial participant pool. We will therefore aim to recruit a sample of 900 cancer survivors, which is a conservative proportion (30%) of the pool of potential participants.

A sample of 900 patients will be sufficient to detect small differences (β=.14) in time intervals between persons seeking care overseas and seeking care on island with a power of 83% assuming a 2-sided α risk of 5% and a statistical 10 covariates in the model. For sensitivity analyses, we will need to restrict the sample to more recently diagnosed participants to minimize problems with recall. Our sampling methods for the pilot study in Saint Lucia yielded 61% of patients diagnosed within 5 years among the total number of cancer survivors recruited. Hence, a sample of 550 survivors (61% of a cohort of 900) with identical parameters will be sufficient to detect medium differences (as low as β=.39) in time intervals. For aim S2, we will use a subsample for these analyses. On the basis of self-reported data from the pilot study in Saint Lucia, 67% of patients completed treatment. Therefore, 603 are expected for the subsample (aim S2). The sample will be sufficiently powered to detect moderate effect sizes (proportionality ratio=1.7, 80% power, and 2-sided 5% alpha risk) using a multivariate model (G*power software, version 3.1.9.4).

### Qualitative Study on Social Support

#### Design

We will use a qualitative descriptive design to gain a deep understanding of the lived experience of cancer survivors relative to social support within the context of transnational cancer care (aim P2). We will study the role of social support in cancer care in Caribbean SIDS using focus groups and one-on-one interviews of informal caregivers and patients. We will encourage participation from patients diagnosed within the last year for better recall. One-on-one interviews will be held with 24 cancer survivors across the 6 islands.

We will approach survivors according to medical travel status. We will interview survivors treated exclusively on the island (n=12), treated partially overseas (n=6), and survivors treated entirely overseas (n=6). Focus groups will capture the synergistic relationships among participants from the 6 islands and enable immediate cross-country comparisons. The caregivers of patients will also be invited to participate and interviewed separately from the survivors. Caregivers will be recruited using means similar to patients: that is, advertisement and leveraging the networks of support groups. Six focus groups consisting of 6 to 9 survivors per caregiver will be conducted (n=54). Members of the focus group may be distinct from those in interviews. Members will have the same medical travel status. The total sample sizes are comparable to other similar qualitative studies [40,55,56].

We will purposefully identify [57] cancer survivors and caregivers of survivors who satisfy the following criteria: (1) are receiving or have already finished their active treatment at the time of the study and (2) received a treatment requiring the intervention of a medical professional.

#### Collection of Qualitative Data

*One-on-one interviews* will be initiated by an introductory question, “Describe the support you received during the cancer journey,” then guided by questions and prompts “who,” “what was the relationship to you? such as a family, friend, neighbors,” and “when.” Participants will be encouraged to focus on aspects of the day-to-day logistics and travel arrangements and experience living overseas. We anticipate that qualitative interviews will last between 1 and 2 hours and will be audio recorded using Zoom. Interviews will be transcribed by investigators using the live transcription feature in Zoom [58]. Qualitative interviews will be organized and scheduled 6 months following the start of the quantitative study. A member of our team with qualitative research experience will moderate the *focus group* in English using a semistructured, open-ended focus group interview guide [59], while a second co-investigator will take notes. Six focus groups in total (3 for survivors and 3 for caregivers). Each country will contribute 3 to 4 participants maximum per focus group (6‐9 persons). Discussions will be held at community centers or cancer support group buildings or held virtually using a suitable video-conferencing tool (eg, MS Teams, Zoom).

Our focus group questions will follow the model proposed by Ruff et al [60], using open-ended questions that will include introductory questions, key questions, and a concluding question. Introductory questions (eg, “Can you talk about the process leading up to your cancer treatment?”) will encourage participants to respond from their own experiences. Key questions will focus on administrative procedures, travel logistics, and lived experience in the country(ies) where they received treatment, with questions such as, “What was it like leaving your home country for treatment?,” “How did you feel about leaving your family and loved one back home?,” and “What were some of changes you experienced when you arrived in the destination country for treatment (e.g. change in climate)?” We will ask concluding questions to ensure that complete information had been obtained. At the end, we will ask, “Is there anything else you would like to say about being in a foreign country for cancer treatment?” Multimedia Appendix 1 shows the draft interview guide for the qualitative study.

#### Qualitative Data Analysis

We will use an inductive thematic analysis approach to identify and interpret patterns or themes in our data. Transcript coding will be data driven through an open coding process [61]. Transcripts will be verified against audio recordings by listening to them back in their entirety. Transcripts will be read repeatedly to ensure familiarity with the data and generate codes. Codes will be grouped into potential themes that capture key aspects of participants’ experiences with overseas cancer care. Two of our authors independently will analyze and code the same sample of responses. We will write analytic memos throughout to capture the thoughts of the interviewers and document emerging relationships within the data. We will apply constant comparison across transcripts to iteratively refine codes and themes. Coding and theme development will continue until thematic saturation is achieved. Furthermore, the decisional criteria that will emerge in the previous focus groups will be reported to the subsequent ones at the end of discussions, with the intention of introducing new insights and data [62]. We will perform member checking to determine if our preliminary findings accurately reflect their experiences as cancer survivors and caregivers. Relevant documents encountered during interviews were examined, such as private insurance policies, ministry guidelines for travel assistance to triangulate with data from interviews. NVivo 10.0 software will be used for theme coding. 62

### Integration of Data and Joint Displays

We will integrate data using joint displays that will allow us to synthesize quantitative findings and directly juxtapose them with themes emerging from interviews and focus groups. This method will contribute to the generation of integrated interpretations that link observed patterns in overseas travel for care to lived experience and identify areas of convergence or complementarity.

### Ethical Considerations

This protocol was approved by McGill University’s Institutional Review Board (ID: A10-M79-24A). Ethics approval was also granted by the Antigua and Barbuda Institutional Review Board (ID: AL-01/102024-ANUIRB), Ethics committees of the Commonwealth of Dominica (ID: not applicable; January 27, 2025), St. George’s University IRB (Grenada, ID: 23005), the Research Ethics Committee of the Medical and Dental Council (Saint Lucia; ID: not applicable; May 2, 2023), the Ministry of Health Ethics Review Committee of St. Kitts (MOH-ERC-2024-09-072), and the National Research Ethics Committee of Saint Vincent and the Grenadines (IRB: MOHWE07062024). The data presented in the pilot were derived from the DCAP study, which was approved by the Research Ethics Committee of the Medical and Dental Council (Saint Lucia; ID: not applicable; April 1, 2019). The study will be conducted in compliance with Good Clinical Practice Procedures and the principles of the Declaration of Helsinki. Participants who meet the eligibility criteria and who wish to take part in either surveys or qualitative interviews will be informed about the study procedures and asked to provide their written informed consent.

Measures will be implemented to ensure the confidentiality and privacy of participant data. All data will be deidentified prior to analysis, and unique study identifiers will be used in place of personal information. Data will be stored securely on password-protected servers, with access restricted to authorized members of the research team. Given the multicountry nature of the study and the small population sizes in participating islands, particular attention will be paid to minimizing risks of deductive disclosure. Reporting of results will follow appropriate data suppression rules for small cell sizes to ensure that individuals cannot be identified. Data sharing between sites will follow agreed protocols to ensure compliance with local regulations and ethical standards.

Participants will not receive financial incentives for participation; however, we will compensate patients with nonmonetary tokens of appreciation in accordance with local ethical guidelines. In addition, where applicable, reasonable accommodations will be provided for patients, such as payment of transportation and refreshments.

### Project Team and Governance

**Figure 4.**
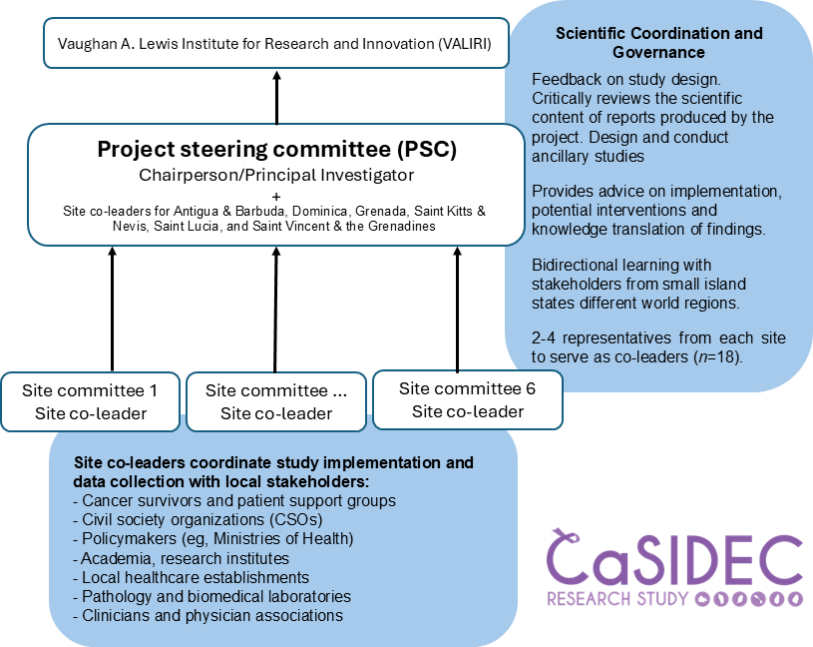
Project governance for the CaSIDEC study. The diagram illustrates the organizational framework, including the Project Steering Committee (PSC) for scientific oversight and site committees responsible for local implementation and stakeholder engagement across participating countries. VALIRI is the study organizer. The CaSIDEC logo is included for identification purposes only. Its reuse does not imply endorsement or affiliation. CaSIDEC: Cancer in small island developing states of the Eastern Caribbean; CSO: civil society organization; VALIRI: Vaughan A. Lewis Institute for Research and Innovation (registered and based in Saint Lucia).

The CaSIDEC study is led by an interdisciplinary team with expertise in epidemiology, clinical oncology, and global health. The principal investigator (AA) is an Epidemiologist from Saint Lucia. He is currently an Assistant Professor at McGill University as well as a researcher and member of the leadership of the Vaughan A. Lewis Institute for Research and Innovation, a multidisciplinary research institute established by the Sir Arthur Lewis Community College in Saint Lucia. All members of the CaSIDEC Study Group, including the Project Steering Committee (PSC), are Afro-Caribbean nationals originally from the OECS member states included in this study protocol. Our group comprises the 18 members of the PSC of the CaSIDEC study and a project manager. In terms of skill set, the PSC comprises 7 researchers, 4 cancer survivors, 4 specialist clinicians, representatives from 6 cancer support groups, and 3 senior technical representatives from the Ministries of Health. Our team composition reflects an intentional commitment to conducting ethically grounded global health research that is locally led. University students also rotate with the study group to provide punctual support in implementation and logistics. Figure 4 shows details of the governance structure for the CaSIDEC study.

## Results

Ethics approvals have been obtained across all 6 participating countries. Planning and development phases have started. Indeed, country-level logistics and preparation began on February 17, 2023, and will continue through December 31, 2026, as we develop partnerships and capacity to execute the medical record abstraction and qualitative interviews across the 6 countries. Test surveys with eligible cancer survivors were conducted from September 2024, and we have recruited 113 cancer survivors as of May 31, 2026 (Antigua: n=3, Dominica: n=9, Grenada: n=9, Saint Kitts: n=20, Saint Lucia: n=20, and Saint Vincent: n=52). We estimate that participant recruitment and data collection will be completed on March 31, 2029. The final results of the CaSIDEC study are expected within 2 years of data collection. The principal outcome of this protocol will be to establish a coordinated multicountry framework for research on cancer survivorship in SIDS, grounded in the needs of the local communities and stakeholders. This work will enable future locally led empirical investigations in this understudied context.

## Discussion

### Comparison With Prior Work

The proposed research is innovative because it addresses a potential determinant of cancer mortality and survivorship specific to a much-understudied population [11,63]. The status quo, as it pertains to research on overseas travel for cancer care, is studies focusing on patients from large low- and-middle-income countries. Furthermore, these studies draw data largely from self-reports from HCPs, clinical registries, and government sponsorship programs. Thus, these studies did not address patient outcomes, their views, or quality of care. Only recently have patient-centered outcomes been investigated among patients from SIDS [5,33,40].

We will investigate, for the first time, overseas travel for cancer care across multiple Caribbean SIDS. We will focus on major knowledge gaps; notably, care pathways, social support, and cancer survivorship, including lifestyle management. The data from this proposal are expected to drive the development of strategies to optimize the overseas referral process, taking into account the psychological well-being of patients and caregivers. Ultimately, in SIDS, cancer survivorship may be improved through shorter delays for treatment, more subsidized care, better mechanisms for travel approval and arrangements, and additional social support at key moments.

### Limitations

Several sources of bias are anticipated as this study is based on a cross-sectional sample of cancer survivors based on retrospective data collection. There are no population-based cancer registries in the OECS to facilitate sampling. Hence, an important limitation of this study will be that we are unable to use random sampling or recruit solely incident cases. This may introduce selection bias that skews our sample toward long-term survivors and patients who would have had less severe disease. In addition, there is potential for recall bias, particularly for self-reported dates for treatment and experiences of care overseas.

To mitigate these limitations, we anticipate a few measures. In addition to soliciting volunteers through communication campaigns, we will systematically recruit patients with newly diagnosed cancer via oncology clinics and membership in local cancer support groups and associations. In terms of handling recall bias, data obtained from medical records will be used to complement self-reported information. These limitations will be considered in the interpretation of results. While recall bias is a limitation when including prevalent cases, sensitivity analyses restricted to those diagnosed within 12 months will reduce this risk.

With an approximate 3000 prevalent cases across the 6 islands [2], our target sample of 900 is feasible (30% of the total pool of potential participants). Hence, the first 3 years will allow for adequate recruitment to achieve reasonably powered analyses, despite potential study interruptions. We anticipate that 2 years will be needed to consolidate data from all countries, clean and prepare the final dataset for analysis, and disseminate. Ample time is needed during the study period to carefully produce dissemination outputs for both international scientific audiences and local stakeholders. The knowledge translation component will take time and may require adequate planning to execute events and actions at critical periods during the year for maximum engagement (eg, October for breast cancer awareness month).

### Knowledge Translation and Future Interventions

Our work will produce recommendations to fill performance gaps in the systems for cancer care in the OECS that will improve patients’ lives at multiple phases. It is also an important first step to developing data resources for high-quality research in understudied SIDS.

Our PSC plans to inform change in practice and policy [64]. We will implement a robust dissemination and knowledge translation strategy rooted in community organizing principles. One of the outputs to be considered will be OECS-wide treatment guidelines recommending destinations based on parameters such as the patient-centered outcomes and waiting times reported from our study. CaSIDEC’s Steering Committee (Figure 4) will work in concert with policymakers to ensure actionable improvements in cancer care. Our strategy includes using the Caribbean Cancer Portal [65] to disseminate our research findings, share best practices, and provide real-time updates to survivors, health care professionals, and policymakers. We will foster interdisciplinary dialogue on critical issues such as treatment delays, diet, PA, and social support systems during or after cancer via a series of conferences, seminars, webinars, and workshops. Our PSC will meet regularly to discuss the advancement in science with local stakeholders [64]. We have also integrated knowledge users and health policy experts in Fiji to further strengthen our capacity for knowledge translation. Their involvement will encourage cross-pollination with practices in other small island contexts and help to emerge innovative concepts.

## Supplementary material

10.2196/90930 Multimedia Appendix 1 Draft interview guide for the CaSIDEC study (Cancer in small island developing states of the Eastern Caribbean) developed by the principal investigator (version dated May 29, 2026). The final version is to be developed in collaboration with the Project Steering Committee.

10.2196/90930 Checklist 1 ObsQual and SPIRIT checklists.

## Abbreviations

CaSIDEC: Cancer in small island developing states of the Eastern Caribbean
DAG: directed acyclic graphs
DCAP: Description of the CAncer health services: diagnosis and treatment Pathways
GIRC: geographically isolated and resource constrained
HCP: health care provider
OECS: Organization of Eastern Caribbean States
PA: physical activity
PSC: Project Steering Committee
SIDS: small island developing states
SPIRIT: Standard Protocol Items: Recommendations for Interventional Trials
SQUASH: Short Questionnaire to Assess Health-Enhancing Physical Activity
TNM: tumor, node, and metastasis
