# Supplementary material for: Overseas Care and Cancer Survivorship in Small Island Developing States of the Eastern Caribbean: Protocol for a Multicountry Mixed Methods Study (CaSIDEC Study)
Source: JMIR Res Protoc. 2026 Jul 31;15:e90930. doi: 10.2196/90930 (PMC13427067; doi:10.2196/90930)
Supplement: Multimedia Appendix 1 [file resprot-v15-e90930-s001.pdf]

Appendix 1

This is a Multimedia Appendix to a full manuscript published in the JMIR Research Protocols.
For full copyright and citation information see http://dx.doi.org/10.2196/jmir.90930

Draft interview guide for the CaSIDEC study

This interview guide is a draft document dated the 29th May 2026. It will be used to study the lived experience of cancer survivors in the CaSIDEC study. It was developed by Dr Aviane Auguste (Principal Investigator). The intention is to ensure comprehensive data collection on social support during the cancer journey, contributing to the broader understanding of patient experiences in the Caribbean SIDS.

I. FRONT MATTER

Purpose

This interview guide is designed to explore the social support experiences of individuals during their cancer journey. The questions are intended to gather detailed insights into the types of support received, the relationships involved, and the timing of this support. The interviews will also explore areas such as day-to-day logistics, travel arrangements, and experiences of living overseas which are not covered by the structured questionnaire (Aim P1),

Sample opening Statement

Thank you for participating in this interview. The purpose of this discussion is to understand the types of support you received during your cancer journey. We are particularly interested in learning about the people who supported you (or the person you supported), the nature of their relationship to you, and when this support was provided. Additionally, we would like to hear about your experiences including your day-to-day logistics and travel arrangements.

Consent

Before we begin, we want to ensure that you understand the purpose of this interview and that your participation is voluntary. The interview will be audio-recorded using Zoom, and all recordings will be transcribed. We will use pseudonyms to protect your identity and the identities of any individuals you mention. Participants will give their written informed consent before proceeding with the study.

Data collected systematically

Basic sociodemographic characteristics will be ascertained as part of both interviews and focus groups process, covering participant background characteristics (e.g. age, gender, marital status, education, private health insurance).

II. IN-DEPTH (ONE-ON-ONE) INTERVIEW

1. Introductory question:

- Describe the support you received during your cancer journey.

a) Guiding Prompts:
   - Who provided this support?
   - What was their relationship to you? (e.g., family, friends, neighbors)
   - When was this support provided?

2. Exploratory questions:
   - Can you describe any day-to-day logistics and travel arrangements that were necessary during your treatment?
   - Did you have to move overseas for your treatment? If so, what was that experience like?
3. Concluding question:
   - Is there anything else you would like to share about your experiences or the support you received during your cancer journey?

III. FOCUS GROUPS

Focus group composition

- Moderator: Principal Investigator
- Co-Moderator: Co-Investigator (Notetaker)

Three focus groups: each consisting of 6–9 survivors/caregivers from across the 6 Caribbean Small Island Developing States (SIDS).

Location

Community centers, cancer support group buildings, or virtually via a video-conferencing tool (e.g. Microsoft Teams, Zoom).

Questions:

1. Introductory questions:
   - Can you talk about the process leading up to your cancer treatment?
2. Key questions:
   - What was it like leaving your home country for treatment?
   - How did you feel about leaving your family and loved ones back home?
   - What were some of the changes you experienced when you arrived in the destination country for treatment (e.g., change in climate)?
3. Concluding question:
   - Is there anything else you would like to say about being in a foreign country for cancer treatment?

Adaptations

Questions will be adjusted according to participants' medical travel status.

Logistics: Interviews will be scheduled six months following the start of the survey from Aim P1.
