# Supplementary material for: Overseas Care and Cancer Survivorship in Small Island Developing States of the Eastern Caribbean: Protocol for a Multicountry Mixed Methods Study (CaSIDEC Study)
Source: JMIR Res Protoc. 2026 Jul 31;15:e90930. doi: 10.2196/90930 (PMC13427067; doi:10.2196/90930)
Supplement: Checklist 1 [file resprot-v15-e90930-s002.docx]

**Supplementary materials**

This is a Multimedia Appendix to a full manuscript published in the JMIR Research Protocols. For full copyright and citation information see http://dx.doi.org/10.2196/jmir.90930

**Title:** Overseas Care and Cancer Survivorship in Small Island Developing States of the Eastern Caribbean (CaSIDEC): Protocol for a multi-country mixed-methods study”

**Contents**

**Table S1:** Observational study protocol checklist (Cross-sectional, Case-control and cohort study design) with educational elements.

**Table S2:** Qualitative study protocol checklist with educational elements.

**Table S3:** SPIRIT 2025 checklist of items to address in a randomized trial protocol*

**Table S1:** Observational study protocol checklist (Cross-sectional, Case-control and cohort study design) with educational elements.

| Item | Item no. | Explanation | Page number in the study protocol. (Use NA for not applicable) |
| --- | --- | --- | --- |
| Title | 1 | Indicate the study’s design with a commonly used term in the title. (if study design not mentioned but title is informative enough to determine the study design) E.g., 1. COVID-19 pandemic brings a sedentary lifestyle in young adults: a cross-sectional and longitudinal study. E.g.,2. Prevalence of dyslipidemias in the Mexican national health and nutrition survey 2006 | 1 |
| Introduction |  |  |  |
| Background/rationale | 2 | Explain the scientific background and rationale for the investigation being reported. This section requires references from current literature. | 3-7 |
| Empty Cell | 3 | The study is necessary in comparison with current evidence. It is important to state the importance of this study in the absence of evidence. If evidence is already available to address the study objectives, consider other objectives of study that addresses the knowledge gap. | 7-8 |
| Objectives | 4 | State specific objectives, including any prespecified hypotheses An objective should be SMART (Specific, Measurable, Achievable, Realistic, Time-bound). E.g., To determine the effect of sex on sepsis-related ICU admission and survival for up to 3-years. | 9-10 |
| Methods |  |  |  |
| Study design | 5 | Present key elements of study design early in the paper A one paragraph of overview of the study design. E.g., We conducted a retrospective cohort study of Australian ICU admissions between January 1, 2018 and December 31, 2020 using data from the Australia and New Zealand Intensive Care Society (ANZICS) Adult Patient Database (APD). … | 10 |
| Setting | 6 | Describe the setting, locations, and relevant dates, including periods of recruitment, exposure, follow-up, and data collection This section may be an independent heading or as a second paragraph in the method section. Some information might have already been described in the first paragraph (overview of study design). Describe the details as if the readers can replicate the study. | 13 |
| Participants | 7 | *Cohort study*—Give the eligibility criteria, and the sources and methods of selection of participants. Describe methods of follow-up Eligibility/selection criteria are designed to address inclusion or exclusion of participants with certain characteristics (factors) that may influence the analysis. It is a method used to control confounding factors. E.g. if a factor such as young age or a gender can affect the outcome, specifically inclusion or exclusion can control the confounding factors. Method of follow-up to be described e.g. by diary, phone call, attendance to the clinic. Mention the time of follow-up (monthly, yearly, etc). The personnel that conduct the follow-up and materials used to record the data. The same description is also applicable for database analysis. Hence, understanding of how the data was collected in the database is important for the description. | NA |
|  | 8 | *Case-control study*—Give the eligibility criteria, and the sources and methods of case ascertainment and control selection. Give the rationale for the choice of cases and controls Case-control study is used mainly for rare or low prevalence of disease where large population could not be easily recruited. Same as above Item 7 explanation on eligibility criteria in cohort study section. E.g., case ascertainment could be from a list of patients eligible in the study and the random sampling/selection method is used. (Please state the types of sampling e.g., simple random sampling, cluster sampling, convenient sampling, etc. Random/probability sampling is preferred) Rationale of the selection of control that similar to case is important to allow fair, unbiased and unconfounded comparison. E.g. healthy control derived from the same setting of the control where they are recruited, similar gender ratio, etc. | NA |
|  | 9 | *Cross-sectional study*—Give the eligibility criteria, and the sources and methods of selection of participants Same as above Item 7 explanation on eligibility criteria in cohort study section. | 11-12 |
|  | 10 | *Cohort study*—For matched studies, give matching criteria and number of exposed and unexposed Matching is a technique to allow match to allow comparisons between two participants who may vary in one key characteristic. Matching allows for control of standard confounding factors such as age and gender. | NA |
|  | 11 | *Case-control study*—For matched studies, give matching criteria and the number of controls per case Same as above Item 10. | NA |
| Variables | 12 | Clearly define all outcomes, exposures, predictors, potential confounders, and effect modifiers. Give diagnostic criteria, if applicable E.g. Participant sex was defined based on the sex recorded in the APD. It is not necessary to specify explicitly which variable is confounding factor and effect modifier. They can be mentioned in discussion after interpreting the results. | 15-16 |
| Data sources/ measurement | 13 | For each variable of interest, give sources of data and details of methods of assessment (measurement). Describe comparability of assessment methods if there is more than one group This section may be combined with above Item 12 when defining the variable. | 16 |
| Bias | 14 | Describe any efforts to address potential sources of bias Provide rationale of certain methods used to address potential bias. E.g., Stratified random sampling was used to prevent imbalance of gender being sampled. | 25-26 |
| Study size | 15 | Explain how the study size was arrived at Sample size is usually calculated and to be stated. If it is not, there should be a clear indication of why the sampling is not required such that all participants in the dataset will be included. For a pilot/feasibility study, they are meant to assess feasibility of certain aspect of the study before conducting a full study (where no literature that can be used to calculate sample size). | 19-20 |
| Quantitative variables | 16 | Explain how quantitative variables will be handled in the analyses. If applicable, describe which groupings will be chosen and why E.g., the gender is treated as an independent variable. Further stratified analysis will be performed for gender. | 15-16 |
| Statistical methods | 17 | Describe all statistical methods, including those used to control for confounding The methods such as T-test, Chi-square, regression, etc must be stated. To control for confounding factors in statistics, a multivariable model is often used i.e. multiple linear/logistic regression, Cox proportional hazard. | 17 |
|  | 18 | Describe any methods used to examine subgroups and interactions E.g., Interactions can be employed within multiple regression models and subsequently, subgroup analysis will be performed on those statistically significant interactions. | 18 |
|  | 19 | Explain how missing data will be addressed Missing data is almost always present, and it should be planned on how to deal with. E.g. exclude missing data if less than 20 %. Intent-to-treat by “worst-case best-case scenario”, multiple imputation, etc. | 18 |
|  | 20 | *Cohort study*—If applicable, explain how loss to follow-up will be addressed This can be addressed by design and by statistics. By design, it should be written in the Item 10 (bias). E.g. participant will be contacted and reason for loss of follow-up is recorded. By statistics, the method as above Item 19. | NA |
|  | 21 | *Case-control study*—If applicable, explain how matching of cases and controls will be addressed Matching with one or more factors reduces the variances and thus, paired T-test, Wilcoxon signed-rank test, McNemar’s test or conditional logistic regression. | NA |
|  | 22 | *Cross-sectional study*—If applicable, describe analytical methods taking account of sampling strategy Depending on the sampling strategy e.g., simple random sampling does not require any specific analytics. However, Multistage cluster sampling requires multilevel analysis such as linear mixed model. | NA |
|  | 23 | Describe any sensitivity analyses If applicable, sensitivity analysis is performed to demonstrate a possible scenario that could exist. E.g., inclusion of a variable and excluding it using the same statistical model. This can be compared. | 18 |
| Appendices |  |  |  |
| Biological specimens | 24 | Plans for collection, laboratory evaluation, and storage of biological specimens for genetic or molecular analysis in the current trial and for future use in ancillary studies, if applicable E.g., The daily blood samples were obtained either separately by the investigator or concurrently with the managing health practitioner/s. The blood samples were collected using ethylenediaminetetraacetic acid tube. The blood samples were centrifuged, and the plasma was obtained. The plasma was transported in an ice box until it could be stored at − 20 degree Celsius (°C). The blood samples were stored at 4 °C if the test was performed within 24–48 h. | NA |

Checklist taken from: Low GK, Subedi S, Omosumwen OF, Jiee SF, Devkota S, Shanmuganathan S, Doyle Z. Development and validation of observational and qualitative study protocol reporting checklists for novice researchers (ObsQual checklist). Eval Program Plann. 2024 Oct;106:102468. doi: 10.1016/j.evalprogplan.2024.102468. Epub 2024 Jul 18. PMID: 39029287.

**Table S2:** Qualitative study protocol checklist with educational elements.

| Item | Item no. | Explanation | Page number in the study protocol. (Use NA for not applicable) |
| --- | --- | --- | --- |
| Title | 1 | (*a*) Indicate the study’s design with a commonly used term in the title. (if study design not mentioned but title is informative enough to determine the study design, please mark as ‘yes’) E.g. The nurse’s experience of decision-making processes in missed nursing care: A qualitative study | 1 |
| Theoretical framework |  |  |  |
|  | 2 | The study is necessary in comparison with current evidence. It is important to state the importance of this study in the absence of evidence. If evidence is already available to address the study objectives, consider other objectives of study that addresses the knowledge gap | 20 |
| Aims/objectives | 3 | What is the purpose/aims and objectives of the study? An objective should be SMART (Specific, Measurable, Achievable, Realistic, Time-bound). E.g. To explore nurses’ perspectives regarding the decision-making processes that lead to missed nursing care and to identify the personal and contextual attributes involved in these processes. | 9-10 |
| Methodological orientation and Theory | 4 | What is the methodological orientation that underpins the study? e.g. grounded theory, discourse analysis, ethnography, phenomenology, content analysis E.g. A phenomenological approach was applied to explore areas about which little is known or to gain an understanding of specific areas. Phenomenology is the study of subjective experience, feelings and behaviours of people | 20 |
| Participant selection |  |  | 11, 19-21 |
| Sampling | 5 | How will participants be selected? e.g. purposive, convenience, consecutive, snowball E.g. A total of 26 nurses were recruited through purposeful sampling | 11-12, 19-21 |
| Description of sample | 6 | What are the important characteristics of the sample? Who are you targeting for inclusion in the study and why? E.g. Nurses are active in various settings and every setting has its specific dynamics. By gaining insight into their perspectives, we were able to compare possibly different views. | 11-12, 19-21 |
| Method of approach | 7 | How will study participants be approached? e.g. face-to-face, telephone, mail, email E.g. Each focus group discussion was led by two researchers. One researcher facilitated the interview, and the other had an observing role and monitored the process. | 11-12, 19-21 |
| Sample size | 8 | How many participants are anticipated being included in the study? | 19 |
| Setting |  |  |  |
| Setting of data collection | 9 | Where will the data be collected? e.g. home, clinic, workplace | 21 |
| Presence of non-participants | 10 | Who will be present during the data collection activities? Anyone besides the participants and researchers? Same as example in Item 7. | 21 |
| Data collection and analysis |  |  |  |
| Interview guide | 11 | What questions will you ask? Can you provide a full question guide, including prompts and guides? Has the interview guide been pilot tested? Or will it be? Or was it derived from available literature? | 21 |
| Data recording | 12 | How will the qualitative data be recorded? e.g. audio recording, video recording, note taking, forms? | 21-22 |
| Field notes | 13 | Will field notes be made? Will they be included for data analysis purposes? | 22 |
| Duration | 14 | What is the anticipated duration of the interviews or focus group discussions? | 21 |
| Qualitative analysis | 15 | Types of analysis that can be used: content analysis, thematic analysis, narrative analysis, discourse analysis, etc. | 22 |
| Member checking | 16 | Do you anticipate returning data or results to participants for accuracy and/or validation purposes? If none, please state as NA. | 22 |

Checklist taken from: Low GK, Subedi S, Omosumwen OF, Jiee SF, Devkota S, Shanmuganathan S, Doyle Z. Development and validation of observational and qualitative study protocol reporting checklists for novice researchers (ObsQual checklist). Eval Program Plann. 2024 Oct;106:102468. doi: 10.1016/j.evalprogplan.2024.102468. Epub 2024 Jul 18. PMID: 39029287.

**Table S3:** SPIRIT 2025 checklist of items to address in a randomized trial protocol*

| **Section / Topic** | **No** | **SPIRIT 2025 checklist item description** | **Reported on page no.** |
| --- | --- | --- | --- |
| **Administrative information** | | |  |
| Title and structured summary | 1a | Title stating the trial design, population, and interventions, with identification as a protocol | 1 |
|  | 1b | Structured summary of trial design and methods, including items from the World Health Organization Trial Registration Data Set | 1 |
| Protocol version | 2 | Version date and identifier |  |
| Roles and responsibilities | 3a | Names, affiliations, and roles of protocol contributors | 1, 29 |
|  | 3b | Name and contact information for the trial sponsor |  |
|  | 3c | Role of trial sponsor and funders in design, conduct, analysis, and reporting of trial; including any authority over these activities | NA |
|  | 3d | Composition, roles, and responsibilities of the coordinating site, steering committee, endpoint adjudication committee, data management team, and other individuals or groups overseeing the trial, if applicable | 29 |
| **Open science** | | |  |
| Trial registration | 4 | Name of trial registry, identifying number (with URL), and date of registration. If not yet registered, name of intended registry |  |
| Protocol and statistical analysis plan | 5 | Where the trial protocol and statistical analysis plan can be accessed | 17-19 |
| Data sharing | 6 | Where and how the individual de-identified participant data (including data dictionary), statistical code, and any other materials will be accessible | 30 |
| Funding and conflicts of interest | 7a | Sources of funding and other support (e.g., supply of drugs) | 31 |
|  | 7b | Financial and other conflicts of interest for principal investigators and steering committee members | 31 |
| Dissemination policy | 8 | Plans to communicate trial results to participants, healthcare professionals, the public, and other relevant groups (e.g., reporting in trial registry, plain language summary, publication) | 26-27 |
| **Introduction** | | |  |
| Background and rationale | 9a | Scientific background and rationale, including summary of relevant studies (published and unpublished) examining benefits and harms for each intervention | 3-7 |
|  | 9b | Explanation for choice of comparator | NA |
| Objectives | 10 | Specific objectives related to benefits and harms | 9-10 |
| **Methods: Patient and public involvement, trial design** | | |  |
| Patient and public involvement | 11 | Details of, or plans for, patient or public involvement in the design, conduct, and reporting of the trial | 29 |
| Trial design | 12 | Description of trial design including type of trial (e.g., parallel group, crossover), allocation ratio, and framework (e.g., superiority, equivalence, non-inferiority, exploratory) | 10-11 |
| **Methods: Participants, interventions, and outcomes** | | |  |
| Trial setting | 13 | Settings (e.g., community, hospital) and locations (e.g., countries, sites) where the trial will be conducted | 13-14 |
| Eligibility criteria | 14a | Eligibility criteria for participants | 11 |
|  | 14b | If applicable, eligibility criteria for sites and for individuals who will deliver the interventions (e.g., surgeons, physiotherapists) | NA |
| Intervention and comparator | 15a | Intervention and comparator with sufficient details to allow replication including how, when, and by whom they will be administered. If relevant, where additional materials describing the intervention and comparator (e.g., intervention manual) can be accessed | NA |
|  | 15b | Criteria for discontinuing or modifying allocated intervention/comparator for a trial participant (e.g., drug dose change in response to harms, participant request, or improving/worsening disease) | NA |
|  | 15c | Strategies to improve adherence to intervention/comparator protocols, if applicable, and any procedures for monitoring adherence (e.g., drug tablet return, sessions attended) | NA |
|  | 15d | Concomitant care that is permitted or prohibited during the trial | NA |
| Outcomes | 16 | Primary and secondary outcomes, including the specific measurement variable (e.g., systolic blood pressure), analysis metric (e.g., change from baseline, final value, time to event), method of aggregation (e.g., median, proportion), and time point for each outcome | 17-18 |
| Harms | 17 | How harms are defined and will be assessed (e.g., systematically, non-systematically) | NA |
| Participant timeline | 18 | Time schedule of enrollment, interventions (including any run-ins and washouts), assessments, and visits for participants. A schematic diagram is highly recommended (see Figure) | 10-11 |
| Sample size | 19 | How sample size was determined, including all assumptions supporting the sample size calculation | 19-20 |
| Recruitment | 20 | Strategies for achieving adequate participant enrollment to reach target sample size | 11 |
| **Methods: Assignment of interventions** | | |  |
| Randomization: |  |  | NA |
| Sequence generation | 21a | Who will generate the random allocation sequence and the method used | NA |
|  | 21b | Type of randomization (simple or restricted) and details of any factors for stratification. To reduce predictability of a random sequence, other details of any planned restriction (e.g., blocking) should be provided in a separate document that is unavailable to those who enroll participants or assign interventions | NA |
| Allocation concealment  mechanism | 22 | Mechanism used to implement the random allocation sequence (e.g., central computer/telephone; sequentially numbered, opaque, sealed containers), describing any steps to conceal the sequence until interventions are assigned | NA |
| Implementation | 23 | Whether the personnel who will enroll and those who will assign participants to the interventions will have access to the random allocation sequence | NA |
| Blinding | 24a | Who will be blinded after assignment to interventions (e.g., participants, care providers, outcome assessors, data analysts) | NA |
|  | 24b | If blinded, how blinding will be achieved and description of the similarity of interventions | NA |
|  | 24c | If blinded, circumstances under which unblinding is permissible, and procedure for revealing a participant’s allocated intervention during the trial | NA |
| **Methods: Data collection, management, and analysis** | | |  |
| Data collection methods | 25a | Plans for assessment and collection of trial data, including any related processes to promote data quality (e.g., duplicate measurements, training of assessors) and a description of trial instruments (e.g., questionnaires, laboratory tests) along with their reliability and validity, if known. Reference to where data collection forms can be accessed, if not in the protocol | 14-16 |
|  | 25b | Plans to promote participant retention and complete follow-up, including list of any outcome data to be collected for participants who discontinue or deviate from intervention protocols |  |
| Data management | 26 | Plans for data entry, coding, security, and storage, including any related processes to promote data quality (e.g., double data entry; range checks for data values). Reference to where details of data management procedures can be accessed, if not in the protocol | NA |
| Statistical methods | 27a | Statistical methods used to compare groups for primary and secondary outcomes, including harms | 17-18 |
|  | 27b | Definition of who will be included in each analysis (e.g., all randomized participants), and in which group | 17-18 |
|  | 27c | How missing data will be handled in the analysis | 18 |
|  | 27d | Methods for any additional analyses (e.g., subgroup and sensitivity analyses) | 18 |
| **Methods: Monitoring** | | |  |
| Data monitoring committee | 28a | Composition of data monitoring committee (DMC); summary of its role and reporting structure; statement of whether it is independent from the sponsor and funder; conflicts of interest and reference to where further details about its charter can be found, if not in the protocol. Alternatively, an explanation of why a DMC is not needed | 23-24, 29 |
|  | 28b | Explanation of any interim analyses and stopping guidelines, including who will have access to these interim results and make the final decision to terminate the trial | 29 |
| Trial monitoring | 29 | Frequency and procedures for monitoring trial conduct. If there is no monitoring, give explanation | NA |
| **Ethics** | | |  |
| Research ethics approval | 30 | Plans for seeking research ethics committee/institutional review board approval | 23 |
| Protocol amendments | 31 | Plans for communicating important protocol modifications to relevant parties | 29 |
| Consent or assent | 32a | Who will obtain informed consent or assent from potential trial participants or authorized proxies, and how | 24 |
|  | 32b | Additional consent provisions for collection and use of participant data and biological specimens in ancillary studies, if applicable | NA |
| Confidentiality | 33 | How personal information about potential and enrolled participants will be collected, shared, and maintained in order to protect confidentiality before, during, and after the trial | 24 |
| Ancillary and post-trial care | 34 | Provisions, if any, for ancillary and post-trial care, and for compensation to those who suffer harm from trial participation | 24 |

*We strongly recommend reading this checklist in conjunction with the SPIRIT 2025 Explanation and Elaboration and the SPIRIT 2025 Expanded Checklist for important clarifications on all the items. We also recommend reading relevant SPIRIT extensions. See [www.consort-spirit.org](http://www.consort-spirit.org)

Citation: Chan A-W, Boutron I, Hopewell S, Moher D, Schulz KF, et al. SPIRIT 2025 statement: updated guideline for protocols of randomised trials. BMJ 2025;389:e081477. <https://dx.doi.org/10.1136/bmj-2024-081477>

© 2025 Chan A-W et al. This is an Open Access article distributed under the terms of the Creative Commons Attribution License (<https://creativecommons.org/licenses/by/4.0/>), which permits unrestricted use, distribution, and reproduction in any medium, provided the original work is properly cited.
